# Fixation-related saccadic inhibition in free viewing in response to stimulus saliency

**DOI:** 10.1038/s41598-022-10605-1

**Published:** 2022-04-22

**Authors:** Oren Kadosh, Yoram S. Bonneh

**Affiliations:** School of Optometry and Vision Science, The Mina & Everard Goodman Faculty of Life Sciences, Bar-Ilan University, Ramat-Gan, Israel

**Keywords:** Saccades, Visual system

## Abstract

Microsaccades that occur during fixation were studied extensively in response to transient stimuli, showing a typical inhibition (Oculomotor Inhibition, OMI), and a later release with a latency that depends on stimulus saliency, attention, and expectations. Here, we investigated the hypothesis that in free viewing every saccade provides a new transient stimulation that should result in a stimulus-dependent OMI like a flashed presentation during fixation. Participants (N = 16) freely inspected static displays of randomly oriented Gabor texture images, with varied contrast and spatial frequency (SF) for periods of 10 s each. Eye tracking recordings were divided into epochs triggered by saccade landing (> 1 dva), and microsaccade latency relative to fixation onset was computed (msRT). We found that the msRT in free viewing was shorter for more salient stimuli (higher contrast or lower SF), as previously found for flashed stimuli. It increased with saccade size and decreased across successive saccades, but only for higher contrast, suggesting contrast-dependent repetition enhancement in free viewing. Our results indicate that visual stimulus-dependent inhibition of microsaccades also applies to free viewing. These findings are in agreement with the similarity found between event-related and fixation-related potentials and open the way for studies combining both approaches to study natural vision.

## Introduction

Visual active exploration of the environment can be obtained by performing continuous eye movements, termed saccades, which are guided by high-level goals and stimulus-driven mechanisms. However, high spatial resolution details are accrued during the stationary interval between successive saccades (“fixations”) by miniature involuntary eye movements including microsaccades and random walk-like ocular drift. Microsaccades during fixation^1–3^ were found to prevent retinal image fading^4,5^. However, they are very similar to larger saccades when fixation locus correction and precise exploration of small regions are required^6^. Saccades and microsaccades are generated by similar neural activity in the superior colliculus (SC)^7^. Other cortical regions are also known to be involved in saccade planning, including the lateral intraparietal area and the frontal eye field^8–11^. The latter was found to be necessary for microsaccade execution after inhibition induced by transients^12^. Moreover, a recent study showed that microsaccades can also be driven by task-relevant goals^6,13^.

The microsaccade rate, typically one or two per second, is modulated by stimulus presentation. Microsaccades are first inhibited and then released with a latency that depends on the stimulus properties, spatial attention, and anticipation^14–18^. Similar temporal dynamics was shown when larger saccades were delayed by irrelevant stimuli^19–22^. It has been suggested that saccadic inhibition is a general “freeze effect”^23^ or Oculomotor Inhibition (OMI), not specific to saccades. This claim is supported by evidence for eye-blink inhibition^24^, catchup saccade inhibition during smooth pursuit, and pursuit inhibition^25^, all of which show similar stimulus-dependent inhibition in response to flashed stimuli, with a similar inhibitory time course^25–27^. For example, in response to a flashed Gabor patch with regular and predictable timing (1 Hz), both the inhibition onset and release times were found to be modulated by the spatial frequency and the contrast of the Gabor, and were found to be correlated with the psychophysical contrast detection threshold (Ref.^28^, Fig. 4). However, it remains unclear whether OMI can be applied to ocular drift. Since our past preliminary evidence^29^ might reflect a pupil measurement artifact, recent evidence suggest an opposite effect of increased drift triggered by a randomly presented unanticipated flash in monkeys^30^. Furthermore, enhancement rather than inhibition was found for the pursuit and catch-up saccades by a superimposed flash in the pursuit direction^31^. Further research is needed to resolve these issues.

Event-related responses constitute the vast majority of the psychophysical, electrophysiological, and eye-tracking studies, assuming that these responses reflect the building blocks of perception. Accumulating evidence from recent free viewing electrophysiological studies suggests that the brain responses following a saccade, termed Fixation-related Potentials (FRP), exhibit electrophysiological components very similar to ERPs in response to transient stimuli. In the current study, we investigated whether fixation-related OMI in natural vision is equivalent to the event-related OMI using flashed stimuli. We investigated the hypothesis that, since every saccade provides fresh input to the visual system, the processing of that input will depend on the post-saccadic stimulus salience. This will be reflected in the timing of subsequent microsaccades, providing a useful means to employ analyses associated with flash-induced saccadic inhibition under the natural viewing conditions tested in the current study. To test this hypothesis, observers were engaged in free viewing of Gabor texture images with various contrast and spatial frequencies presented for long durations. We found intriguing similarities between flash-induced saccadic inhibition properties and the fixation durations after saccades in free viewing.

## Methods

### Participants

Twenty-two observers in total, 11 females and 11 males, ages 22–40 participated in the experiments. Seventeen were recruited for Experiment 1 (contrast): 9 females and 8 males; one male participant was removed from the data analysis of this experiment due to the low quality of the data (50% bad data). Sixteen participants were recruited for experiment 2 (spatial frequency): 6 females and 10 males. In summary, each experimental group consisted of 16 participants, 11 of whom participated in both experiments. All participants had normal or corrected-to-normal vision and were naïve to the purpose of the study, except for the first author. The experiments were approved by the Bar-Ilan University Internal Review Board (IRB) ethics committee. All participants gave written informed consent and all the experiments were conducted according to the IRB guidelines.

### Apparatus

We used our standard experimental setup from all our previous studies, including the Eyelink 1000 plus (SR Research) eye tracker with a sample rate of 500 Hz, a 100 Hz calibrated 24-in FHD LCD monitor (Eizo Foris fg2421), and the in-house-developed integrative stimulus presentation and analysis tool (PSY) developed by Y.S. Bonneh. Stimuli were displayed at a distance of 0.6 m. We used a 35 mm lens positioned 0.52 m from the participant’s stabilized head using a chin and forehead rest. Both experiments were administered in dim light and the screen background was gray with 50 cd/m^2^ luminance. All recordings were done binocularly, with analyses performed on data from the left eye. A standard 9-point calibration was performed before each session.

### Stimuli and procedures

**Figure 1:**
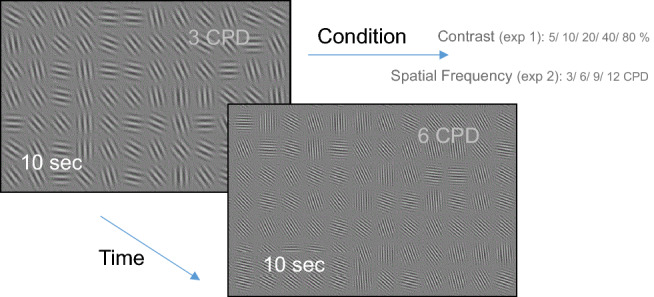
Visual stimuli and the experimental paradigm. Observers free viewed a sequence of full-screen texture images of random orientation Gabor patches for 10-s trial periods. In two separate experiments, the contrast (Exp1) and the spatial frequency (Exp2) were varied across trials in random order.

Observers were instructed to freely inspect the full-screen static displays of Gabor textures in a random orientation for ten-second presentation periods (trials, see Fig. 1). Trials under different conditions were mixed in random order; each observer completed 5 × 2 min runs of 3 trials per condition. The texture contrast varied across conditions in the first experiment (5, 10, 20, 40, and 80% and 6 CPD) and the textures’ spatial frequency varied in experiment 2 (3, 6, 9, 12 CPD, and 50% contrast). Each texture contained Gabor patches with a 0.5° Gaussian envelope, organized in a 20 × 15 matrix, with a 0.6° distance between centers. Eye movements were recorded, and fixation-related epochs were calculated by saccade landing times.

### Data analysis

#### Saccade and microsaccade detection

For saccade detection, we used the algorithm introduced by Engbert and Kliegl^15^; it is based on eye movement velocity, and it has been implemented in our recent study^32^. Raw data were first smoothed using the LOWESS method with a window of 15 ms to optimize microsaccade extraction, especially for noisy recordings^15^. Microsaccades were detected as movements exceeding 8 SD of mean velocity in 2D velocity space, as in Refs.^32,33^. A velocity range of 8°/s–150°/s, an amplitude range of 0.08°–1°, and a minimum duration of 9 ms were allowed for the microsaccades. Eye blinks were detected as in our previous study^24^; we first detected periods with zero (or undefined) pupil size, and then extended these periods by estimating the eyes’ closed and open times, based on the vertical eye movement that typically precedes the blink^32^. Periods of missing data within an epoch, for example, during an eye blink, were discarded from the analysis with an additional margin of 50 ms, without discarding the whole epoch. The rejection rate varied across recordings, with a mean rate of 10 ± 8% in the contrast experiment (1) and 5 ± 4% in the spatial frequency experiment (2). One participant was excluded from experiment 1 due to a high rejection rate of over 50%. Epochs were extracted, triggered by the saccade (> 1°) landing time in a range of − 0.2 s to 0.8 s relative to the fixation onset with some overlap between epochs. This was taken into consideration when computing the microsaccade reaction time (msRT).

#### Calculation of the microsaccade rate function

The microsaccade rate modulation function was calculated as in our previous studies^33–35^; this was done mainly to illustrate the time course of microsaccade occurrences without conducting a statistical analysis. The rate was calculated for the raw microsaccade onsets, and it is described here briefly. For each epoch the rate was computed by convolving a raw rate estimate of one microsaccade per sample duration with a Gaussian window having a sigma of 50 ms. The rates were averaged across the epochs within observers separately for each condition, and then averaged across observers, with error bars computed across observers on demeaned (within observer) data (Cousineau and Morey’s method^36^, see also Bonneh et al.^28^).

#### Saccade and microsaccade RT calculation

The Microsaccade Reaction Time (msRT) was calculated for each epoch relative to the fixation onset in a predefined time window, as the latency of the first microsaccade in that window. The time range was selected to accommodate for the inter participant’s variance. Epochs with microsaccades in the relevant interval in both experiments constituted 29% ± 9 SD of all epochs, compared to ~ 85% in our previous study with flashed stimuli^24^. This could be due to the difference in the event rate: 1 Hz in the previous study with flashed stimuli, compared to 1–3 Hz of free viewing fixations (with a mean fixation duration of ~ 0.5 s) in the current experiments. The first fixation per trial was always ignored to avoid the flash effect on the OMI. The microsaccade RTs (msRT) were averaged across the epochs of each condition within observers and then averaged across observers, with error bars computed across observers on demeaned (within observer) data, with a correction factor (multiplied by √ (n/(n − 1)). This method for computing the error bars provides a better representation of within-participant effects (Cousineau and Morey’s method^36^, see also Bonneh et al.^28^). The saccade reaction time (sacRT) was calculated as the time interval between the current fixation onset and the next fixation onset, including only MS-free fixations. The 3D plots in Figs. 3 and 5, illustrating the saccade number and saccade size effects on the ms/sac RT, were smoothed using a mean filter over a rectangle of size 3 × 3. Basically, the element “xi, yi” was replaced by the mean of the rectangle centered on “xi, yi”. Any missing elements were ignored in the averaging.

#### Statistical assessment

Usually statistical analysis of variance (One-way ANOVA) and multiple comparison post-hoc tests were performed using Matlab 2018b. We first verified that the msRT distributions under different conditions came from normal distributions with equal variance. Another statistical method that was used is the Linear Mix Model (LMM)^37^. The responses were fitted to a simple model of maximum likelihood with spatial frequency or contrast used as the predictor variable, and the observer’s variability was set as the random effect. For each case, we computed the standard error (SE), the regression coefficient (b), the t-test (t), and the p-value of the LMM (p_LMM_) value at a 95% confidence level. In addition, we computed Pearson’s linear correlation coefficient (r^2^) for the group averages of each plot.

## Results

*In experiment 1* we investigated whether the fixation-related microsaccade inhibition in free viewing, i.e., the latency of the first microsaccade that follows the large saccade that starts the fixation, depends on the stimulus contrast, as found in previous studies with transient stimuli. To test this, we extracted epochs triggered by fixation onset based on the saccade landing time in a range of − 0.2 s to 0.8 s relative to this onset (see the “Methods” section). Next, we will report the results of the microsaccade reaction time (msRT) for (1) the effect of contrast, (2) the effect of inducing saccade size, and (3) the cumulative effect (facilitation) across serial saccades.

### The effect of contrast

**Figure 2:**
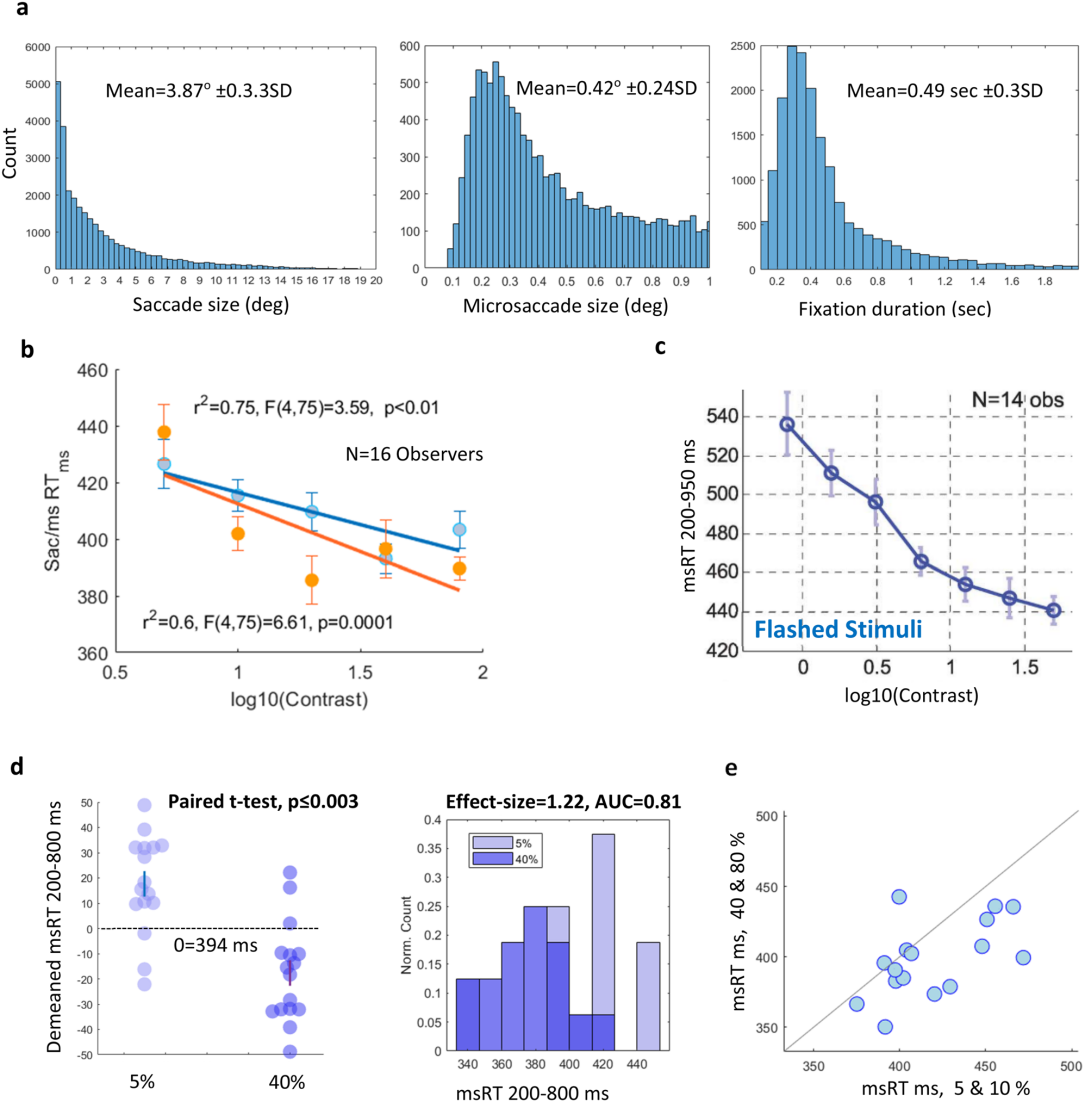
The results for experiment 1: The effect of contrast. (**a**) Frequency histograms for data pooled across all conditions and observers. (Left) A saccade size histogram including microsaccades. (Middle) A microsaccade (< 1 dva) histogram. (Right) A fixation duration histogram (< 2 s). (**b**) A fixation-related msRT and sacRT (calculated over MS-free fixations) as a function of Log contrast, averaged across observers (n = 16), with error bars computed on demeaned (within observer) data with correction (see the “Methods” section). Significance was assessed using One-way Anova, yielding p < 0.01 for microsaccades (< 1 dva) and p = 0.0001 for larger saccades (> 1 dva). (**c**) Results for msRT as a function of Log contrast taken from a previous study with flashed stimuli by Bonneh et al. ^28^ for comparison. (**d**) A comparison between 5 and 40% contrast (left panel) shows longer msRTs for the lower contrast (paired t-test, p ≤ 0.003). Histogram comparisons of 5% and 40% contrast showing a large effect size (1.22 Cohen’s d) and an Area under the curve of the ROC of 0.81 (right panel). (**e**) A scatter plot of the individual observer’s msRT results for low contrast on the X-axes and high contrast on the Y-axes with a symmetry line, showing that almost all observers had longer msRTs for the low contrast.

The results for msRT under various contrast conditions are shown in Fig. 2. Saccade and microsaccade size, as well as fixation duration histograms for all observers and conditions combined are shown in Fig. 2a. The mean saccade and microsaccade sizes were 3.87° ± 3.3SD and 0.42° ± 0.24SD, respectively. We computed the msRT and sacRT (microsaccade/saccade reaction time, see the Methods), as the first microsaccade or saccade occurrence from 200 ms after the fixation onset. Figure 2b shows msRT and sacRT as a function of log_10_(Contrast); it shows a negative linear relation (r^2^ = 0.75 for microsaccades and r^2^ = 0.6 for larger saccades, Pearson’s correlation). Statistical assessment by One-way Anova yielded F(4,75) = 3.59, p < 0.01 and F(4,75) = 6.61, p = 0.0001 for microsaccades and saccades, respectively. Figure 2c shows the results for msRT in response to a presentation of a single transient Gabor grating with various contrast settings from a previous study by Bonneh et al.^24^. These results show a similar trend of shorter latencies in response to higher contrast. Figure 2d shows a significant difference between msRT in 5% and 40% contrast (p ≤ 0.003, Paired t-test) and a histogram comparison shows a large effect size estimation (E-size = 1.22, Cohen’s d) and the Area under the curve (AUC = 0.81) of the ROC. The scatter plot in Fig. 2e shows that most participants had longer microsaccade latencies under low contrast conditions (5%, 10% combined), compared with high contrast (40%, 80% combined).

### The effects of successive saccades and saccade size (exp 1)

**Figure 3:**
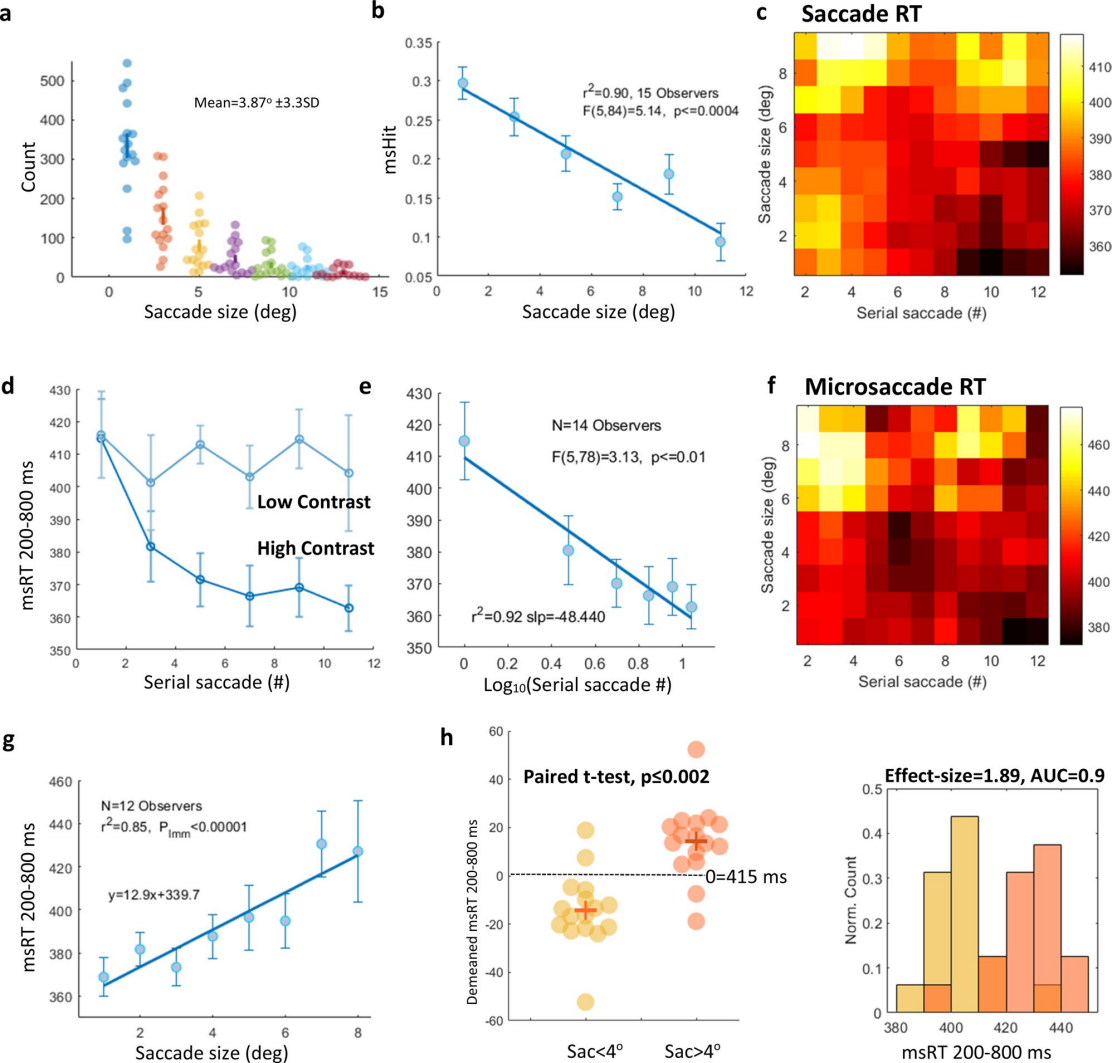
The results for experiment 1: Facilitation over repeated saccades and the saccade size. (**a**) A saccade size histogram with a dot per participant. (**b**) The microsaccade hit rate (msHit) as a function of saccade size (deg), the significance assessed using One-way Anova, F(5,84) = 5.14, p ≤ 0.0004. (**c**) 2D smoothed (see the “Methods” section) fixation-related sacRT (MS-free fixations); all conditions combined as a function of the serial saccade number over time (X-axes) and the saccade size (Y-axes). Light and dark colors represent longer and shorter RTs, respectively. (**d**) Fixation-related msRT as a function of the serial saccade number for high (dark blue) and low contrast, with error bars denoting 1SE computed on the demeaned data, n = 14 (see the “Methods” section). (**e**) msRT as a function of Log contrast only for significance assessment using LMM (see the “Methods” section), p ≤ 0.001. (**f**) The same as c for msRT. (**g**) Fixation-related msRT under all conditions as a function of saccade size (deg) with LMM assessment yielding significance, slope = 12.95, t(94) = 4.8, p ≤ 0.00001 with CI (7.6, 18.3). (**h**) A comparison between msRT in trials triggered by small saccades (< 4°) and large saccades (> 4°), p ≤ 0.002, paired t-test (left panel). msRT histogram comparisons for small and large saccades, showing a very large effect size (1.89 Cohen’s d) and AUC = 0.9 (the right panel).

To gain a deeper understanding of the free-viewing process, we analyzed two properties that are likely to modulate the fixation-triggered effects: (1) the size of the inducing saccade and (2) the cumulative effect of successive saccades. To investigate these effects, we analyzed msRT as a function of saccade size (the distance in degrees) and of the serial saccade number within a trial, throughout the 10-s periods of stimulus presentation within a trial; the results are presented in Fig. 3. Figure 3a describes the occurrences of saccade size using a frequency histogram; each dot represents a participant. Figure 3b shows the microsaccade hit rate (msHit), computed as the percent of epochs containing microsaccades within a time range, which is similar to the selected range for the msRT calculation, as a function of saccade size (deg). It shows a decrease in msHit with saccade size. Figure 3c,f show in a 3D plot a decrease in sac/msRT over successive saccades (darker colors in the lower right corner) and an increase as a function of the saccade distance (light colors in the upper left corner) with trials from all observers and conditions combined. The results were 2D smoothed (see the “Methods” section) to emphasize the similar spatio-temporal dependency of the saccade and microsaccade RTs.

The decrease in msRT over time for high contrast is clearer in Fig. 3d (darker blue), showing a fast decline in the first four saccades, reaching a plateau after the fourth saccade. Significance for this effect was assessed by One-way Anova (F(5,78) = 3.13, p ≤ 0.01) with 14 observers contributing data, shown in Fig. 3e. Two observers were excluded due to the lack of data points for the latest saccades (high serial numbers). Note that after approximately 10 saccades the number of occurrences is significantly reduced due to the mean number of fixations per trial for all participants and conditions, which was ~ 9 ± 5 SD. In comparison to high contrast, the results for msRT under the low contrast conditions (5 and 10%), denoted in light blue in Fig. 3d_,_ do not show a decline in the msRT over successive saccades (or time).

The msRT as a function of the saccade size is shown in Fig. 3g. There is an increase in msRT with increased saccade distance (slope = 12.95, t(94) = 4.8, p_LMM_ ≤ 0.00001 with CI (7.6,18.3)). We further tested the significance of the saccade size effect on the msRT by categorizing saccades as either small saccades (1–4°) or large saccades (4°–20°). The 4° criterion was based on the average saccade size of 3.87° ± 3.3SD. Figure 3h compares msRT as a function of small and large saccades, p ≤ 0.002, paired t-test with a very large effect size, ES = 1.89, Cohen’s d and AUC = 0.9.

**Figure 4:**
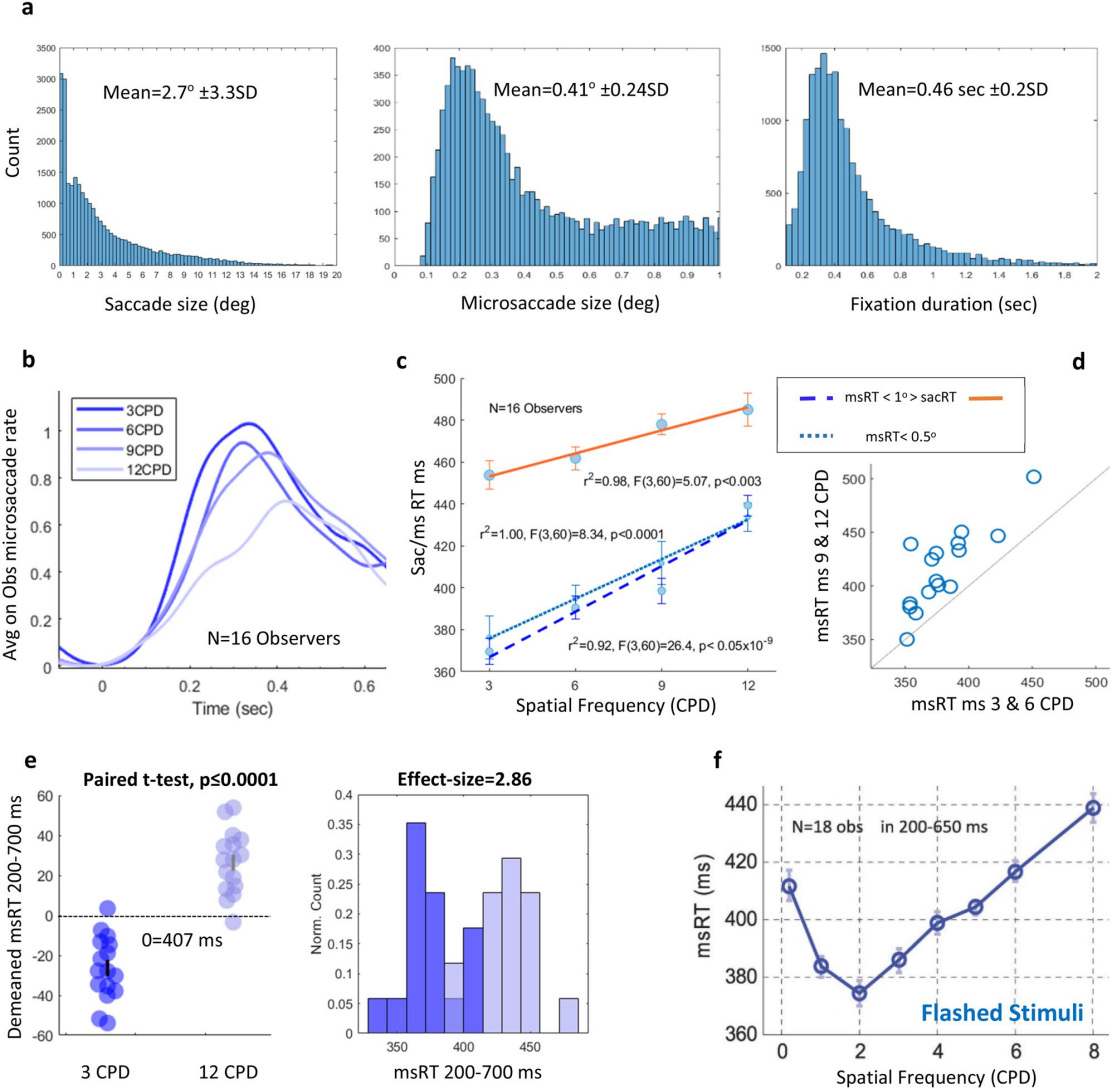
The results for experiment 2: The effect of spatial frequency. (**a**) The frequency histograms under all conditions and for all observers combined. (Left) A saccade size histogram including microsaccades. (Middle) Microsaccades up to 1° histogram. (Right) Fixation duration up to a 2 s histogram. (**b**) The fixation-related MS rate per second averaged across observers (n = 16). (**c**) The fixation-related msRT and sacRT (calculated over MS-free fixations) as a function of SF averaged across observers (n = 16), with 1SE error bars computed on the demeaned (see the “Methods” section), all showing a similar positive linear trend. msRT (< 1°, dashed blue line), r^2^ = 0.92, F(3,60) = 26.4, p < 0.05 × 10^–9^, One-way Anova. msRT (< 0.5°, dotted light blue line), r^2^ = 1, F(3,60) = 8.34, p < 0.0001, One-way Anova. sacRT (> 1°, orange line ), r^2^ = 0.98, F(3,60) = 5.07, p < 0.003, One-way Anova. (**d**) A diagonal scatter plot of the individual observer’s msRT results for a low SF on the X-axes and high SF on the Y-axes, showing that most observers are above the diagonal line indicating longer msRTs for the high SF. (**e**) A comparison between the two extremes; 3 and 12 CPD show longer msRTs for the higher SF, p ≤ 0.0001, paired t-test (left panel). Histogram comparisons of 3 and 12 CPD showing a huge effect size, ES = 2.86, Cohen’s d (right panel). (**f**) The results for msRT as a function of spatial frequency taken from a previous study of flashed stimuli for comparison^28^, with a notable similarity in the relevant SF range.

*In experiment 2* we investigated whether the fixation-related microsaccade latency in free viewing depends on the stimulus spatial frequency (SF), as found in previous studies with transient stimuli. Fixation-related epochs were created as in experiment 1. Saccade and microsaccade size, as well as the fixation duration frequency histograms for all observers and conditions combined are shown in Fig. 4a. Observers (N = 16) freely inspected full-screen images of Gabor textures with random orientations for ten seconds for each trial. The SF of the Gabor patches varied from 3 to 12 CPD between conditions and the trials from different conditions were mixed.

### The effect of spatial frequency and microsaccade size

First, we calculated the microsaccade rate modulation function, averaged across observers (see the Methods) for the different spatial frequencies (3, 6, 9, and 12 CPD). Figure 4b shows microsaccade inhibition and release following a saccade with a timing that depended on the texture SF. We calculated the saccade reaction time (sacRT) for saccades larger than 1° (MS-free fixations only, see the “Methods” section) and computed the msRT (microsaccade reaction time, see the Methods), as the first microsaccade released from inhibition after the fixation onset. To test for a possible effect of the microsaccade size, we analyzed small (< 0.5°) and larger (< 1°) MS separately. Figure 4c presents the msRT (< 1° and < 0.5°) and sacRT as a function of the spatial frequency, showing a positive linear relation. msRT (< 1°), r^2^ = 0.92, Pearson’s correlation, F(3,60) = 26.4, p < 0.05 × 10^–9^, One-way Anova. msRT (< 0.5°), r^2^ = 1, Pearson’s correlation, F(3,60) = 8.34, p < 0.0001, One-way Anova. sacRT (> 1°), r^2^ = 0.98, Pearson’s correlation, F(3,60) = 5.07, p < 0.003, One-way Anova. The individual scatter plot in Fig. 4d shows that all participants except one had longer microsaccade latencies with high SF (9 and 12 CPD combined), compared with low SF (3 and 6 CPD). Figure 4e shows a difference between msRT in 3 and 12 CPD with a histogram comparison and an effect size estimation using Cohen’s d. The Paired t-test shows a significant difference, p ≤ 0.0001 with a huge effect size. Figure 4f shows the results for msRT in response to a presentation of a single transient Gabor grating in different SFs from a previous study by Bonneh et al.^24^. These results show a similar trend of increased latencies together with SF.

### The effects of successive saccades and the saccade size (exp 2)

**Figure 5:**
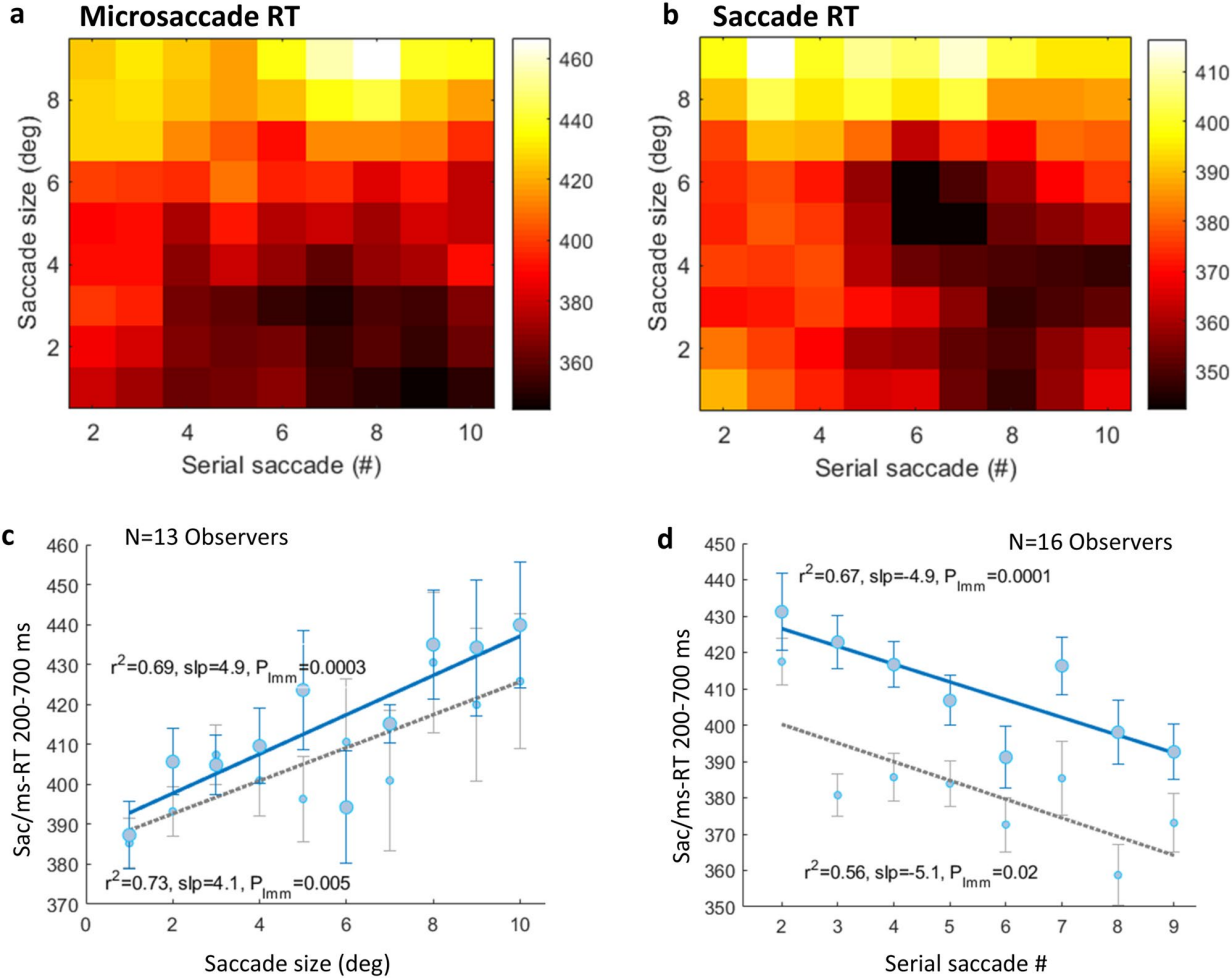
The results for experiment 2: Facilitation over repeated saccades and the saccade size. A fixation-related microsaccade (**a**) and saccade (from MS-free fixations) and (**b**) RTs as a function of the serial saccade number over time (X-axes) and saccade size (Y-axes). Light and dark colors represent longer and shorter RTs, respectively. 2D smoothing was applied (see the “Methods” section) and all conditions were combined for a clearer demonstration of the similar trend for msRT and sacRT. (**c**) A fixation-related saccade (blue)/microsaccade (gray) latency (sac/ms RT) as a function of saccade distance (deg), averaged across observers (n = 13) with 1SE error bars computed on the demeaned data (see the “Methods” section). Significance was assessed using LMM (see the “Methods” section). b(slope) = 4.9, t(118) = 3.77, p ≤ 0.0003 with CI (2.3, 7.5); b(slope) = 4.1, t(118) = 2.8, p ≤ 0.005 with CI (1.2, 7), for sacRT and msRT, respectively. (**d**) A fixation-related saccade (blue)/microsaccade (gray) latency (sac/ms RT) as a function of the serial saccade number, averaged across observers (n = 16) with 1SE error bars computed on the demeaned data (see the “Methods” section). Significance was assessed using LMM (see the “Methods”); b(slope) =  − 4.9, t(126) =  − 3.9, p ≤ 0.0001 with CI (− 7.3, − 2.4); b(slope) =  − 3, t(118) =  − 2.2, p ≤ 0.02 with CI (− 5.7, − 0.3), for sacRT and msRT, respectively.

We computed sacRT and msRT as a function of saccade size (the distance in degrees) and the serial saccade number over time within a single trial as in experiment 1. Figure 5 shows similar effects of saccade size and the serial saccade number, on saccade and microsaccade RTs, all conditions combined. It demonstrates, using a 2D smoothed 3D plot (see the “Methods” section), a decrease in msRT (a) and sacRT (b) over successive saccades reflected by the darker colors in the lower right corner; and an increase as a function of the saccade distance reflected by the lighter colors at the upper left corner of the plot. A positive relation is observed in Fig. 5c, r^2^ = 0.69 for sacRT and r^2^ = 0.73 for msRT (Pearson’s correlation), as a function of saccade size, yielding a significant LMM analysis (see the “Methods” section) to assess the linear trend; b(slope) = 4.9, t(118) = 3.77, p ≤ 0.0003 with CI (2.3, 7.5); b(slope) = 4.1, t(118) = 2.8, p ≤ 0.005 with CI (1.2, 7), respectively. Three observers were excluded due to a lack of data points for large saccade sizes. A negative relation is observed in Fig. 5d, r^2^ = 0.67 for sacRT and r^2^ = 0.56 for msRT, as a function of the serial saccade number yielding significance using LMM; b(slope) =  − 4.9, t(126) =  − 3.9, p ≤ 0.0001 with CI (− 7.3, − 2.4); b(slope) =  − 3, t(118) =  − 2.2, p ≤ 0.02 with CI (− 5.7, − 0.3), respectively.

## Discussion

We investigated the possible application of the saccadic inhibition phenomenon to free viewing, assuming that the transient stimulus is induced by a saccade that triggers a refractory period. We investigated the properties of this refractory period in terms of the microsaccade/saccade RT and its dependence on the fixated stimulus saliency, specifically on the stimulus contrast and the spatial frequency (SF). Our main finding is that a fixation-related refractory period in free viewing follows the same principles as the event-related OMI with transient stimuli. We replicated two known phenomena from event-related experiments: (1) the dependence on stimulus saliency and (2) modulation by stimulus repetition^38^. Taken together, these data suggest that the ocular refractory period in free viewing is similar to the event-related OMI; thus, a new fixation is like a flashed stimulus, which provides fresh input to the visual system and induces OMI depending on that stimulus and some additional factors. Next, we discuss the main findings of the study: (1) the effect of stimulus saliency (contrast and spatial frequency), (2) the effect of accumulation or repetition facilitation across successive saccades, (3) the effect of saccade size, and (4) the similarities and differences between free viewing and transient stimuli.

### The effect of stimulus saliency

Our results from both experiments show that saccade and microsaccade RT, which reflects the OMI duration following the inducing saccade, decreases as a function of contrast (Fig. 2) and increases as function of SF (Fig. 4); this corresponds to a shorter stimulus processing time with growing saliency and reduced sensory noise. These results suggest that the notion of oculomotor inhibition (OMI) may be applied to free viewing, with a similar dependency on contrast and SF as found for flashed stimuli^28^. Our results are compatible with those of previous findings reported by Bonneh et al.^28^, with a similar change in msRT (release microsaccade inhibition) for changes in contrast and spatial frequency, as shown when comparing Figs. 2b,c and 4d,f. Note that the flashed stimulus experiments were conducted with a single vertical Gabor patch with a fixed 2.7° envelope, flashed for 100 ms, whereas our stimuli consisted of static images containing smaller Gabor patches (0.5° envelope) arranged in a 20 × 15 matrix with random orientations.

Our results for spatial frequency (SF) are also consistent with two previous studies that manipulated SF in static displays, one with searching Gabor textures showing longer inter-saccadic intervals for higher SF^39^ and the other with fixation on static Gratings showing longer inter-microsaccade intervals for higher SF^40^; both showed the same trend as our saccade and microsaccade RT results. Longer fixation durations for higher spatial frequency are also predicted by the computational arguments of Ahissar et al.^41^ and Rucci et al.^42^ based on the idea that the slow fixational drift is optimized for higher SF, compared to the saccade transients. According to this argument, the fixation period of accumulating information from the fixational drift, which is longer for higher SF is the main factor that determines the latency of the next saccade or microsaccade.

### The effect of successive saccades: facilitation by repetition

We further investigated the fixation-related microsaccade latency over time, i.e., across successive saccades, as a function of the serial saccade number during a single trial. We expected latencies to decrease as a function of the saccade number, as we found for repeated identical (or predicted) flashed stimuli (Ref.^38^, preliminary data). Interestingly, the current results showed a decreased msRT over successive saccades in the high but not the low contrast trials.

To explain these results, we considered the different models suggested by Grill-Spector et al.^43^ that account for brain responses to repeated stimuli. The first repetition suppression model to consider is the classical “fatigue” effect or contrast adaptation induced by the repeated saccade landing stimulation. For flashed stimuli, the adaptation results in a shift in the contrast response function in V1^44^, which is equivalent to reducing the effective contrast. Since lower contrast was found to increase the OMI duration or the msRT in our current data as well as previously for flashed stimuli^24^, it is reasonable to assume that our finding of an opposite speedup effect is inconsistent with the suppressive or “fatigue” model. Note that a classical contrast adaptation effect that produces a significant reduction in the cortical response requires dozens of repetitions and is primarily found with peripheral stimulation, whereas our data are based on very few foveal stimulations.

The facilitation model is an alternative model that can account for the shortening of the stimulus processing time and the associated OMI^45,46^. The model assumes synaptic potentiation that leads to faster responses^43^ when identification accuracy is at a ceiling, e.g., a shorter microsaccade latency for repeated high contrast stimuli in the current study. With degraded stimuli, as under the low contrast conditions, identification is likely to be less accurate; thus, facilitation is weaker (fewer neurons will drop out in the sharpening process with repetition).

A less profound (p_LMM_ = 0.02, see Fig. 5d) facilitation effect was found for microsaccade latencies in the second experiment with various spatial frequencies that were presented in high contrast (50%); however, a highly significant facilitation by the repetition effect (p_LMM_ = 0.0001, see also Fig. 5d) was found for saccade (> 1°) latencies. The shortening of the sacRT, which is the same as fixation duration, over time could also be related to a shift to the ambient mode of exploration during the trial^47^.

### The effect of saccade size

A novel finding of the current study is the increase in the sac/ms RT with the distance of the saccade in the near periphery. We hypothesized that the microsaccade latency following a large saccade vs. short saccade would be longer because the contribution of the peripheral preview, assumed to speedup processing, is reduced as a function of eccentricity. In both experiments, we found elongation of the fixation-related microsaccade latency in proportion to the saccade size, as hypothesized (see Figs. 3f,g, 5a,c). Microsaccade RT, as a function of saccade size, produced a significant positive linear trend for saccade size <  ~ 10°. Data from larger saccades were excluded in this particular case because they were scarce (see Fig. 3a). We further categorized the saccades into small and large saccades and compared the msRT between categories. We chose a boundary criterion of 4° based on the average saccade size of 3.87° (median = 3°) and by estimating the effect size for larger criteria. The results (Fig. 3h) show a very clear and highly significant effect of a faster msRT for the smaller saccades (an effect size of ~ 1.9).

Taken together, these results support our hypothesis of a reduced contribution of the noisier pre-saccadic extra-foveal peripheral preview for longer saccades. It is also supported by evidence for a benefit or the facilitation effects of a pre-saccadic preview in reading^48^ and in face processing^49,50^.

### Shared saccade and microsaccade characteristics

According to some recent studies, saccades and microsaccades share a common neurophysiological basis and respectively, perform similar functions outside and within the foveal region of the visual field. Both are binocular eye movements with a ballistic nature that follows the main sequence^51^ (Zuber et al.^7^) with related SC neuronal activity that shares the same firing rate properties^7^. This led us to question whether the OMI effects we found for microsaccades also apply to larger saccades. Saccade inhibition is related to activity in SC rostral poles (fixation cells)^52^; it is also responsible for microsaccade generation^53^. Thus, the SC motor map contains desired gaze locations and provides a continuum of motor neurons, from the rostral SC to the caudal, which encode both small saccade vectors and large ones^54,55^. Both are linked to covert attention^10,14,15^ and both can be voluntarily executed, which was shown with memory-guided microsaccades^13^ and for microsaccades executed with ongoing task demands^6^. Moreover, Poletti et al. used small face images ~ 1° in size as targets for microsaccades to show that the scanning strategies within the foveal region were similar to the way that saccades explore a larger face^56^. In our study, the participants initiated saccades (> 1°) and microsaccades (< 1°) to freely explore a Gabor texture scene. We observed lower microsaccade rates in free viewing, up to 30% (see Fig. 3b, compared with our previous study using flashed stimuli^24^ (up to ~ 85%) as was also previously reported^57^. Our results showed that the intervals between microsaccades or saccades from a previous saccade follow a similar pattern of dependency on the stimulus characteristics (see Fig. 4c) and are similar for facilitation across successive saccades and saccade distance effects (see Figs. 3c,f, 5). This advances our understanding of the resemblance in functionality.

### Transient stimuli vs. free viewing: similarities and differences

Whereas past electrophysiological studies typically probed the visual system with flashed stimuli to study the event-related brain responses (ERPs), recent studies measure fixation-related potentials (FRPs) in free viewing, to probe the event-related brain responses induced by saccades under natural viewing conditions (Refs.^58–62^, see also our preliminary FRP results^63^). In these studies, the observer controls the spatial and temporal properties of the stimulus by controlling the saccades, whereas flashed stimuli are often presented at random times and could disrupt the ongoing processing. Nevertheless, the emerging picture is that FRPs are similar to ERPs, although not identical, with evidence including the ERP components of N170 for faces^61^, P1 for checkerboard stimuli^64^, and P300 in visual search^60,65^. In the current study we compared eye movements under event-related and fixation-related conditions (Figs. 2, 4). The event-related data refer to temporally predictable events, with saccadic inhibition onset that may precede the flashed stimuli^66^, and release from inhibition that depended on the stimulus saliency, similar to many other studies of oculomotor inhibition^23,67,68^. Here we show that the fixation-related process in free viewing is similar to the event-related process and that it is similarly stimulus dependent (Figs. 3, 4). For example, following a fixation in free viewing, the latency of the following fixational eye movement (microsaccade < 0.5 dva) is correlated with the spatial frequency of the stimulus (Fig. 4c). We suggest that similar mechanisms are involved in both the event-related and fixation-related conditions, despite whether it is termed oculomotor inhibition, oculomotor freezing, a refractory period, or the processing time.

There is an alternative interpretation of the free viewing results, which does not involve an explicit inhibitory mechanism, and it questions the analogy to transient stimuli. According to this account, in free viewing, the system just plans a sequence of saccades, each with a refractory period caused by the planning of the next saccade, depending primarily on its post-saccadic stimulus (monkey results^39^). However, many studies show that the refractory period depends on the processing of the fixation stimulus, while making ‘stay-or-go’ decisions that reflect competition between foveal and peripheral signals, as described in the LATEST model by Tattler et al.^69^. This is mediated via reciprocal inhibition, where the foveal signals act to “stay”, delaying the onset of the next movement, whereas the peripheral signals act to shorten the fixation^70^. Such inhibitory interactions were found in the monkey SC between foveal (fixational) neurons and saccade buildup neurons^71^. Similar competition is suggested by the results of the ‘gap paradigm’^72^, in which saccade latencies are decreased by adding a gap between the fixation stimulus offset and the peripheral target onset^70,72,73^. In free viewing, additional competition also exists for multiple targets like in the countermanding paradigm^74,75^. Moreover, in the Chen et al. study^39^, the human data are based on a visual search task among an array of Gabor patches, showing a stimulus-dependent inter-saccadic interval similar to our results, which could be related to the post-saccadic as well as to the pre-saccadic stimulus and thus could be in agreement with our explanation. In summary, both free viewing and transient event processing involve similar “stay-or-go” decisions that incorporate inhibitory mechanisms that delay the saccades, also depending on the fixated stimulus; in this sense the two types of processes are similar and possibly involve the same mechanisms.

Finally, we can consider predictions of the shared mechanism hypothesis that could be tested experimentally. Since for flashed stimuli, the microsaccade inhibition is generally related to the processing time of the flashed stimulus, e.g., with word^35^, sound^68,76^, and face^33,77^ categorization, it is possible to generate and test similar conditions in natural vision; it involves moving the eyes over a static display from one stimulus to another. With large enough saccades, which prevent pre-saccadic peripheral identification, the saccadic inhibition or refractory period should depend primarily on the fixated stimulus; it is predicted to be similar to the flashed stimulus results. Our preliminary data from such a paradigm involving a visual oddball in space confirm the hypothesis of the similarity between flashed stimuli and free viewing.

### Summary and conclusions

Our results suggest that the Oculomotor Inhibition (OMI) concept can be applied to free viewing, where every fixation starts with a transient stimulation by a saccade. We found that the latencies of the following microsaccades or saccades were affected by stimulus saliency in a way similar to flashed stimuli. Moreover, saccades and microsaccades were similarly affected by repeating stimulation with decreased latencies over successive saccades and increased latencies with longer saccade distance. These findings add to the accumulating evidence of the relations between event-related and fixation-related processing and open the way for studies combining oculomotor and electrophysiological measures to study natural vision.

## Data Availability

The experimental datasets generated during the current study will be available from the corresponding author upon reasonable request.
